# Appetite

**Published:** 1869-07

**Authors:** 


### APPETITE.

Fussy nurses are an abomination. They are continually making all manner of absurd propositions for the comfort of a patient and are ever annoying the medical attendant with ostentatious questions. Almost every physician has been wrought up to a swearing pitch by the attempts of a nurse to cram his patients with food. "Why, Doctor, she don't eat anything at all. She never *can* keep up long, if she don't eat something. I have given her some rice, some chicken broth, some jelly, some baked apple and some bread and milk, but she don't seem to have one bit of appetite. What had I better prepare for her?" *Nothing*, we reply. Nurse lifts her hands in holy horror, and declares, privately, that it is her opinion that we are starving that patient, for no-body can "keep up" unless they eat.

Now, we desire to remind all such meddlesome creatures that, although food is often better than physic, yet, a physician is supposed to know *when* the one or the other is applicable, and if he does *not* know, the patient's *appetite* will soon apprise him as to whether food is essential to her existence or not.

*Never force a patient to eat.* Food that is crammed down one's throat can do no possible good, but will just as certainly do harm. A very good rule to follow with reference to the feeding of sick-folks is, never to say anything about food until it is inquired for. Then, unless the article selected would be absolutely hurtful, let the sick-one have what her fancy calls for. Nine cases in ten, she will ask for just what is needed, and is suited to her case. Appetite is always the best guide to follow when we are uncertain as to whether any particular article of food is suited to one's stomach or not. If you want it—eat it, and if your appetite is a normal one, it will most likely "agree with you."—But remember not to *dose* sick folks with eatables—nature knows best when food is necessary, and will call for it in due time through the appetite. It is a mistaken idea that a patient will improve more rapidly if they are bountifully-fed. Nature always lays up a store of food in the system, better for supplying the wants of those prostrated by fevers, than anything that can be offered by the stomach.

